# Supplementary material for: Metabolite Changes in Maternal and Fetal Plasma Following Spontaneous Labour at Term in Humans Using Untargeted Metabolomics Analysis: A Pilot Study
Source: Int J Environ Res Public Health. 2019 Apr 30;16(9):1527. doi: 10.3390/ijerph16091527 (PMC6539865; doi:10.3390/ijerph16091527)
Supplement: Supplementary file 1 [file ijerph-16-01527-s001.pdf]

International Journal of
Environmental Research and Public Health

MDPI

Supplementary Materials: Metabolite Changes in Maternal and Fetal Plasma Following Spontaneous Labour at Term in Humans Using Untargeted Metabolomics Analysis: A Pilot Study

Katherine A. Birchenall, Gavin I. Welsh and Andrés López Bernal

Table S1. Fold-change in cord plasma (VC/EC) for metabolites (xenobiotics excluded) with significant *p* (≤0.05) and *q* (≤0.10), ordered according to fold-change (greatest to smallest), with corresponding sub-pathway and super-pathway.

Metabolite	Sub-Pathway	Super-Pathway	Fold-Change (VC/EC)	*p*-Value	*q*-Value
**Heme**	Hemoglobin and Porphyrin Metabolism	Cofactors and Vitamins	**14.30**	0.0239	0.0918
**Fibrinopeptide A, Phosphono-Ser**	Fibrinogen Cleavage Peptide	Peptide	**11.71**	0.0144	0.0689
**Glucuronide Of C10H18O2**	Partially Characterized Molecules	Partially Characterised Molecules	**9.98**	0.0159	0.0734
**Spermidine**	Polyamine Metabolism	Amino Acid	**7.26**	0.0172	0.0764
**Adenosine 5'-Diphosphoribose (Adp-Ribose)**	Nicotinate and Nicotinamide Metabolism	Cofactors and Vitamins	**7.25**	0.0180	0.0784
**Glycerol 3-Phosphate**	Glycerolipid Metabolism	Lipid	**7.13**	0.0064	0.0435
**Glucuronide Of C10H18O2**	Partially Characterized Molecules	Partially Characterised Molecules	**7.07**	0.0214	0.0887
**Inosine**	Purine Metabolism, (Hypo)Xanthine/Inosine containing	Nucleotide	**6.01**	0.0016	0.0194
**Cysteine-Glutathione Disulfide**	Glutathione Metabolism	Amino Acid	**5.13**	0.0035	0.0312
**Cortisol**	Corticosteroids	Lipid	**3.57**	0.0000	0.0015
**Adenosine 3',5'-Cyclic Monophosphate (Camp)**	Purine Metabolism, Adenine containing	Nucleotide	**3.53**	0.0009	0.0141
**Maltose**	Glycogen Metabolism	Carbohydrate	**3.39**	0.0254	0.0925
**Trans-Urocanate**	Histidine Metabolism	Amino Acid	**3.23**	0.0012	0.0148
**Corticosterone**	Corticosteroids	Lipid	**3.09**	0.0002	0.0070
**Cortisone 21-Sulfate**	Corticosteroids	Lipid	**3.02**	0.0001	0.0051
**Hypoxanthine**	Purine Metabolism, (Hypo)Xanthine/Inosine containing	Nucleotide	**2.96**	0.0001	0.0049
**Androstenediol (3Alpha, 17Alpha) Monosulfate**	Androgenic Steroids	Lipid	**2.80**	0.0110	0.0583
**3-Phosphoglycerate**	Glycolysis, Gluconeogenesis and Pyruvate Metabolism	Carbohydrate	**2.77**	0.0233	0.0918
**5Alpha-Androstan-3Alpha,17Beta-Diol Monosulfate**	Androgenic Steroids	Lipid	**2.63**	0.0141	0.0677
**N-Formylphenylalanine**	Tyrosine Metabolism	Amino Acid	**2.56**	0.0056	0.0403
**Xanthine**	Purine Metabolism, (Hypo)Xanthine/Inosine containing	Nucleotide	**2.55**	0.0000	0.0008
**Palmitoleate (16:1N7)**	Long Chain Monounsaturated Fatty Acid	Lipid	**2.52**	0.0190	0.0805
**1,2-Dipalmitoyl-Gpe (16:0/16:0)**	Phosphatidylethanolamine (PE)	Lipid	**2.51**	0.0269	0.0955
**Margaroylcarnitine (C17)**	Fatty Acid Metabolism (Acyl Carnitine, Long Chain Saturated)	Lipid	**2.47**	0.0105	0.0579
**Eicosenoylcarnitine (C20:1)**	Fatty Acid Metabolism (Acyl Carnitine, Monounsaturated)	Lipid	**2.43**	0.0045	0.0362
**5-Dodecenoate (12:1N7)**	Medium Chain Fatty Acid	Lipid	**2.41**	0.0018	0.0209
**3-Hydroxylaurate**	Fatty Acid, Monohydroxy	Lipid	**2.40**	0.0000	0.0020

**S-Adenosylhomocysteine (Sah)**	Methionine, Cysteine, SAM and Taurine Metabolism	Amino Acid	**2.34**	0.0223	0.0898
**Gamma-Glutamylglutamate**	Gamma-glutamyl Amino Acid	Peptide	**2.33**	0.0286	0.0988
**Galactonate**	Fructose, Mannose and Galactose Metabolism	Carbohydrate	**2.31**	0.0185	0.0796
**Androsterone Sulfate**	Androgenic Steroids	Lipid	**2.27**	0.0007	0.0128
**Cysteinylglycine**	Glutathione Metabolism	Amino Acid	**2.21**	0.0123	0.0625
**Lactate**	Glycolysis, Gluconeogenesis and Pyruvate Metabolism	Carbohydrate	**2.13**	0.0000	0.0018
**Alpha-Ketoglutarate**	TCA Cycle	Energy	**2.13**	0.0011	0.0148
**Adenosine 5'-Monophosphate (Amp)**	Purine Metabolism, Adenine containing	Nucleotide	**2.12**	0.0246	0.0918
**Hexadecadienoate (16:2N6)**	Long Chain Polyunsaturated Fatty Acid (n3 and n6)	Lipid	**2.09**	0.0004	0.0094
**Stearoylcarnitine (C18)**	Fatty Acid Metabolism (Acyl Carnitine, Long Chain Saturated)	Lipid	**2.08**	0.0256	0.0925
**Myristoleate (14:1N5)**	Long Chain Monounsaturated Fatty Acid	Lipid	**2.03**	0.0165	0.0745
**3-Hydroxydecanoate**	Fatty Acid, Monohydroxy	Lipid	**2.01**	0.0002	0.0066
**Glycerophosphoinositol**	Phospholipid Metabolism	Lipid	**1.98**	0.0134	0.0653
**(16 Or 17)-Methylstearate (A19:0 Or I19:0)**	Fatty Acid, Branched	Lipid	**1.96**	0.0041	0.0353
**Fructose**	Fructose, Mannose and Galactose Metabolism	Carbohydrate	**1.96**	0.0186	0.0796
**Palmitoleoylcarnitine (C16:1)**	Fatty Acid Metabolism (Acyl Carnitine, Monounsaturated)	Lipid	**1.95**	0.0009	0.0141
**1-Palmitoyl-2-Oleoyl-Gpe (16:0/18:1)**	Phosphatidylethanolamine (PE)	Lipid	**1.93**	0.0095	0.0554
**Oleoylcarnitine (C18:1)**	Fatty Acid Metabolism (Acyl Carnitine, Monounsaturated)	Lipid	**1.90**	0.0010	0.0145
**Laurate (12:0)**	Medium Chain Fatty Acid	Lipid	**1.90**	0.0010	0.0146
**Acetylcarnitine (C2)**	Fatty Acid Metabolism (Acyl Carnitine, Short Chain)	Lipid	**1.89**	0.0004	0.0094
**10-Heptadecenoate (17:1N7)**	Long Chain Monounsaturated Fatty Acid	Lipid	**1.89**	0.0026	0.0282
**Pantothenate**	Pantothenate and CoA Metabolism	Cofactors and Vitamins	**1.89**	0.0241	0.0918
**N-Acetylglucosamine/N-Acetylgalactosamine**	Aminosugar Metabolism	Carbohydrate	**1.88**	0.0033	0.0304
**Malate**	TCA Cycle	Energy	**1.86**	0.0012	0.0148
**Ribulonate/Xylulonate**	Pentose Metabolism	Carbohydrate	**1.85**	0.0009	0.0141
**Cortisone**	Corticosteroids	Lipid	**1.85**	0.0010	0.0145
**Dihomo-Linoleoylcarnitine (C20:2)**	Fatty Acid Metabolism (Acyl Carnitine, Polyunsaturated)	Lipid	**1.84**	0.0059	0.0413
**Oleate/Vaccenate (18:1)**	Long Chain Monounsaturated Fatty Acid	Lipid	**1.84**	0.0067	0.0438
**Linolenate [Alpha Or Gamma; (18:3N3 Or 6)]**	Long Chain Polyunsaturated Fatty Acid (n3 and n6)	Lipid	**1.83**	0.0067	0.0438
**Xanthosine**	Purine Metabolism, (Hypo)Xanthine/Inosine containing	Nucleotide	**1.82**	0.0005	0.0104
**10-Nonadecenoate (19:1N9)**	Long Chain Monounsaturated Fatty Acid	Lipid	**1.81**	0.0012	0.0151
**Linoleate (18:2N6)**	Long Chain Polyunsaturated Fatty Acid (n3 and n6)	Lipid	**1.81**	0.0032	0.0301
**Laurylcarnitine (C12)**	Fatty Acid Metabolism (Acyl Carnitine, Medium Chain)	Lipid	**1.80**	0.0084	0.0518
**Caprate (10:0)**	Medium Chain Fatty Acid	Lipid	**1.79**	0.0031	0.0299
**Succinate**	TCA Cycle	Energy	**1.78**	0.0098	0.0565
**2-Hydroxyglutarate**	Fatty Acid, Dicarboxylate	Lipid	**1.76**	0.0024	0.0275
**Myristate (14:0)**	Long Chain Saturated Fatty Acid	Lipid	**1.75**	0.0071	0.0450
**Estrone 3-Sulfate**	Estrogenic Steroids	Lipid	**1.74**	0.0132	0.0651
**Myristoleoylcarnitine (C14:1)**	Fatty Acid Metabolism (Acyl Carnitine, Monounsaturated)	Lipid	**1.72**	0.0077	0.0483
**Palmitoylcarnitine (C16)**	Fatty Acid Metabolism (Acyl Carnitine, Long Chain Saturated)	Lipid	**1.72**	0.0115	0.0597
**Eicosenoate (20:1)**	Long Chain Monounsaturated Fatty Acid	Lipid	**1.69**	0.0030	0.0298
**Glycerol**	Glycerolipid Metabolism	Lipid	**1.68**	0.0000	0.0003
**N-Acetyltaurine**	Methionine, Cysteine, SAM and Taurine Metabolism	Amino Acid	**1.68**	0.0016	0.0194

**Caprylate (8:0)**	Medium Chain Fatty Acid	Lipid	**1.67**	0.0160	0.0734
**Dihomo-Linoleate (20:2N6)**	Long Chain Polyunsaturated Fatty Acid (n3 and n6)	Lipid	**1.65**	0.0045	0.0362
**Linoleoylcarnitine (C18:2)**	Fatty Acid Metabolism (Acyl Carnitine, Polyunsaturated)	Lipid	**1.64**	0.0003	0.0087
**(14 Or 15)-Methylpalmitate (A17:0 Or I17:0)**	Fatty Acid, Branched	Lipid	**1.64**	0.0029	0.0298
**Uracil**	Pyrimidine Metabolism, Uracil containing	Nucleotide	**1.64**	0.0109	0.0583
**Myristoylcarnitine (C14)**	Fatty Acid Metabolism (Acyl Carnitine, Long Chain Saturated)	Lipid	**1.63**	0.0010	0.0146
**Pyruvate**	Glycolysis, Gluconeogenesis and Pyruvate Metabolism	Carbohydrate	**1.62**	0.0031	0.0298
**(S)-3-Hydroxybutyrylcarnitine**	Fatty Acid Metabolism (Acyl Carnitine, Hydroxy)	Lipid	**1.61**	0.0236	0.0918
**Glutamate, Gamma-Methyl Ester**	Glutamate Metabolism	Amino Acid	**1.59**	0.0287	0.0988
**Decanoylcarnitine (C10)**	Fatty Acid Metabolism (Acyl Carnitine, Medium Chain)	Lipid	**1.58**	0.0131	0.0651
**5-Dodecenoylcarnitine (C12:1)**	Fatty Acid Metabolism (Acyl Carnitine, Monounsaturated)	Lipid	**1.57**	0.0245	0.0918
**Arachidonoylcarnitine (C20:4)**	Fatty Acid Metabolism (Acyl Carnitine, Polyunsaturated)	Lipid	**1.55**	0.0006	0.0105
**Palmitate (16:0)**	Long Chain Saturated Fatty Acid	Lipid	**1.54**	0.0065	0.0435
**Isocitric Lactone**	TCA Cycle	Energy	**1.54**	0.0221	0.0898
**Margarate (17:0)**	Long Chain Saturated Fatty Acid	Lipid	**1.53**	0.0049	0.0373
**5,6-Dihydrouracil**	Pyrimidine Metabolism, Uracil containing	Nucleotide	**1.53**	0.0094	0.0554
**(12 Or 13)-Methylmyristate (A15:0 Or I15:0)**	Fatty Acid, Branched	Lipid	**1.53**	0.0100	0.0570
**1-Stearoyl-2-Oleoyl-Gpi (18:0/18:1)**	Phosphatidylinositol (PI)	Lipid	**1.51**	0.0264	0.0943
**Phenyllactate (Pla)**	Phenylalanine Metabolism	Amino Acid	**1.50**	0.0054	0.0392
**Vanillactate**	Tyrosine Metabolism	Amino Acid	**1.49**	0.0005	0.0099
**Imidazole Lactate**	Histidine Metabolism	Amino Acid	**1.47**	0.0256	0.0925
**Stearoyl-Arachidonoyl-Glycerol (18:0/20:4)**	Diacylglycerol	Lipid	**1.44**	0.0161	0.0734
**Docosadienoate (22:2N6)**	Long Chain Polyunsaturated Fatty Acid (n3 and n6)	Lipid	**1.43**	0.0053	0.0391
**Isocitrate**	TCA Cycle	Energy	**1.43**	0.0202	0.0850
**Creatine**	Creatinine Metabolism	Amino Acid	**1.41**	0.0029	0.0298
**Fumarate**	TCA Cycle	Energy	**1.41**	0.0043	0.0358
**2-Hydroxy-3-Methylvalerate**	Leucine, Isoleucine and Valine Metabolism	Amino Acid	**1.41**	0.0130	0.0650
**Stearate (18:0)**	Long Chain Saturated Fatty Acid	Lipid	**1.31**	0.0068	0.0440
**Glucose**	Glycolysis, Gluconeogenesis and Pyruvate Metabolism	Carbohydrate	**1.31**	0.0105	0.0579
**Oleoyl Ethanolamide**	Endocannabinoid	Lipid	**1.31**	0.0226	0.0904
**Pentadecanoate (15:0)**	Long Chain Saturated Fatty Acid	Lipid	**1.30**	0.0043	0.0358
**5-Oxoproline**	Glutathione Metabolism	Amino Acid	**1.18**	0.0035	0.0312
**Serine**	Glycerine, Serine and Threonine Metabolism	Amino Acid	**0.85**	0.0113	0.0591
**Tryptophan**	Tryptophan Metabolism	Amino Acid	**0.83**	0.0243	0.0918
**Arginine**	Urea cycle, Arginine and Proline Metabolism	Amino Acid	**0.82**	0.0103	0.0579
**Histidine**	Histidine Metabolism	Amino Acid	**0.80**	0.0047	0.0368
**Citrulline**	Urea cycle, Arginine and Proline Metabolism	Amino Acid	**0.80**	0.0213	0.0887
**Gamma-Glutamylleucine**	Gamma-glutamyl Amino Acid	Peptide	**0.80**	0.0257	0.0925
**N1-Methylinosine**	Purine Metabolism, (Hypo)Xanthine/Inosine containing	Nucleotide	**0.79**	0.0275	0.0967
**Indole-3-Carboxylic acid**	Tryptophan Metabolism	Amino Acid	**0.74**	0.0086	0.0526
**Carnosine**	Histidine Metabolism	Amino Acid	**0.73**	0.0232	0.0918
**Gamma-Glutamyl-Alpha-Lysine**	Gamma-glutamyl Amino Acid	Peptide	**0.72**	0.0180	0.0784
**Picolinate**	Tryptophan Metabolism	Amino Acid	**0.69**	0.0047	0.0368

**2'-Deoxyuridine**	Pyrimidine Metabolism, Uracil containing	Nucleotide	**0.69**	0.0251	0.0925
**1-Methylnicotinamide**	Nicotinate and Nicotinamide Metabolism	Cofactors and Vitamins	**0.68**	0.0239	0.0918
**5-Methyluridine (Ribothymidine)**	Pyrimidine Metabolism, Uracil containing	Nucleotide	**0.66**	0.0037	0.0324
**Xanthurenate**	Tryptophan Metabolism	Amino Acid	**0.66**	0.0152	0.0719
**N-Acetyltyrosine**	Tyrosine Metabolism	Amino Acid	**0.62**	0.0053	0.0391
**N1-Methyl-4-Pyridone-3-Carboxamide**	Nicotinate and Nicotinamide Metabolism	Cofactors and Vitamins	**0.60**	0.0286	0.0988
**N1-Methyl-2-Pyridone-5-Carboxamide**	Nicotinate and Nicotinamide Metabolism	Cofactors and Vitamins	**0.56**	0.0110	0.0583
**1-Stearoyl-Gpi (18:0)**	Lysophospholipid	Lipid	**0.54**	0.0170	0.0759
**Isoleucylglycine**	Dipeptide	Peptide	**0.51**	0.0003	0.0081
**N-Acetylkynurenine**	Tryptophan Metabolism	Amino Acid	**0.51**	0.0119	0.0611
**N-Acetyl-2-Aminooctanoate**	Fatty Acid, Amino	Lipid	**0.47**	0.0110	0.0583
**Cholate**	Primary Bile Acid Metabolism	Lipid	**0.44**	0.0221	0.0898
**Valylglycine**	Dipeptide	Peptide	**0.38**	0.0003	0.0081
**3-Dehydrocholate**	Secondary Bile Acid Metabolism	Lipid	**0.29**	0.0001	0.0065
**Palmitoylcholine**	Fatty Acid Metabolism (Acyl Choline)	Lipid	**0.18**	0.0093	0.0554
**Arachidonoylcholine**	Fatty Acid Metabolism (Acyl Choline)	Lipid	**0.08**	0.0029	0.0298

Table S2. Fold-change in maternal (intervillous) plasma (VM/EM) for metabolites (xenobiotics excluded) with significant *p* (≤0.05) and *q* (≤0.10), ordered according to fold-change (greatest to smallest), with corresponding sub-pathway and super-pathway.

Metabolite	Sub-Pathway	Super-Pathway	Fold-Change (VM/EM)	*p*-Value	*q*-Value
**Glucuronide Of C10H18O2**	Partially Characterized Molecules	Partially Characterized Molecules	**10.85**	0.0113	0.053
**Maleate**	Fatty Acid, Dicarboxylate	Lipid	**10.42**	0.0359	0.0857
**Adipate (C6-Dc)**	Fatty Acid, Dicarboxylate	Lipid	**8.06**	0.0178	0.0625
**N-Acetylaspartate (Naa)**	Alanine and Aspartate Metabolism	Amino Acid	**7.14**	0.0007	0.0113
**Caproate (6:0)**	Medium Chain Fatty Acid	Lipid	**6.71**	0.012	0.0539
**Glucuronide Of C10H18O2**	Partially Characterized Molecules	Partially Characterized Molecules	**6.44**	0.0267	0.0759
**Corticosterone**	Corticosteroids	Lipid	**5.41**	0.0001	0.0076
**Cortisol**	Corticosteroids	Lipid	**5.02**	0.0003	0.0079
**1-Oleoylglycerol (18:1)**	Monoacylglycerol	Lipid	**4.49**	0.0069	0.0417
**Arachidoylcarnitine (C20)**	Fatty Acid Metabolism(Acyl Carnitine)	Lipid	**4.24**	0.0128	0.0559
**Androsterone Sulfate**	Androgenic Steroids	Lipid	**3.99**	0.0042	0.0325
**Epiandrosterone Sulfate**	Androgenic Steroids	Lipid	**3.7**	0.0132	0.0565
**Androstenediol (3Alpha, 17Alpha) Monosulfate**	Androgenic Steroids	Lipid	**3.45**	0.0089	0.0476
**Glucuronate**	Aminosugar Metabolism	Carbohydrate	**3.31**	0.0314	0.08
**12-Hete**	Eicosanoid	Lipid	**3.21**	0.0053	0.0357
**5Alpha-Androstan-3Beta,17Beta-Diol Disulfate**	Androgenic Steroids	Lipid	**3.09**	0.0347	0.0839
**Oleoyl-Linoleoyl-Glycerol (18:1/18:2)**	Diacylglycerol	Lipid	**2.93**	0.0407	0.0919
**9,10-Dihome**	Fatty Acid, Dihydroxy	Lipid	**2.9**	0.0091	0.0476
**Lactosyl-N-Nervonoyl-Sphingosine (D18:1/24:1)**	Ceramides	Lipid	**2.83**	0.0024	0.0261
**1-Stearoyl-2-Oleoyl-Gpe (18:0/18:1)**	Phosphatidylethanolamine (PE)	Lipid	**2.81**	0.0211	0.0693

**Androsterone Glucuronide**	Androgenic Steroids	Lipid	**2.74**	0.0138	0.0565
**1-Linoleoyl-Gpi (18:2)**	Lysophospholipid	Lipid	**2.72**	0.0005	0.0109
**Palmitoleoyl-Linoleoyl-Glycerol (16:1/18:2)**	Diacylglycerol	Lipid	**2.7**	0.0358	0.0857
**3-Methyladipate**	Fatty Acid, Dicarboxylate	Lipid	**2.68**	0.0295	0.078
**Eicosenoylcarnitine (C20:1)**	Fatty Acid Metabolism(Acyl Carnitine)	Lipid	**2.58**	0.0003	0.0079
**Erucate (22:1N9)**	Long Chain Fatty Acid	Lipid	**2.58**	0.001	0.0144
**P-Cresol-Glucuronide**	Tyrosine Metabolism	Amino Acid	**2.57**	0.0461	0.0942
**Palmitoyl-Linoleoyl-Glycerol (16:0/18:2)**	Diacylglycerol	Lipid	**2.57**	0.0265	0.0759
**Oleoyl-Linoleoyl-Glycerol (18:1/18:2)**	Diacylglycerol	Lipid	**2.49**	0.0121	0.0539
**2-Hydroxybehenate**	Fatty Acid, Monohydroxy	Lipid	**2.49**	0.0304	0.0792
**Oleoyl-Oleoyl-Glycerol (18:1/18:1)**	Diacylglycerol	Lipid	**2.47**	0.0449	0.0934
**Pristanate**	Fatty Acid, Branched	Lipid	**2.45**	0.0439	0.0934
**Xanthosine**	Purine Metabolism, (Hypo)Xanthine/Inosine containing	Nucleotide	**2.4**	0.0171	0.0615
**Oleoyl-Oleoyl-Glycerol (18:1/18:1)**	Diacylglycerol	Lipid	**2.38**	0.0066	0.0414
**Maltose**	Glycogen Metabolism	Carbohydrate	**2.36**	0.0178	0.0625
**Lignoceroylcarnitine (C24)**	Fatty Acid Metabolism(Acyl Carnitine)	Lipid	**2.35**	0.0044	0.0338
**Ribulonate/Xylulonate**	Pentose Metabolism	Carbohydrate	**2.34**	0.0003	0.0079
**5Alpha-Pregnan-3Beta,20Alpha-Diol Monosulfate (1)**	Progestin Steroids	Lipid	**2.31**	0.0049	0.0353
**12,13-Dihome**	Fatty Acid, Dihydroxy	Lipid	**2.31**	0.043	0.0934
**Fructose**	Fructose, Mannose and Galactose Metabolism	Carbohydrate	**2.28**	0.0326	0.0809
**Diacylglycerol (16:1/18:2, 16:0/18:3)**	Diacylglycerol	Lipid	**2.26**	0.0094	0.0477
**Ximenoylcarnitine (C26:1)**	Fatty Acid Metabolism(Acyl Carnitine)	Lipid	**2.25**	0.0025	0.0261
**5Alpha-Pregnan-3Beta,20Alpha-Diol Monosulfate (2)**	Progestin Steroids	Lipid	**2.25**	0.0039	0.0318
**Eicosenoate (20:1)**	Long Chain Fatty Acid	Lipid	**2.23**	0.002	0.024
**Diacylglycerol (12:0/18:1, 14:0/16:1, 16:0/14:1)**	Diacylglycerol	Lipid	**2.21**	0.0424	0.0934
**Campesterol**	Sterol	Lipid	**2.18**	0.0113	0.053
**1-Palmitoyl-2-Oleoyl-Gpe (16:0/18:1)**	Phosphatidylethanolamine (PE)	Lipid	**2.18**	0.0225	0.0728
**Dihomo-Linoleoylcarnitine (C20:2)**	Fatty Acid Metabolism(Acyl Carnitine)	Lipid	**2.17**	0.0307	0.0792
**Laurate (12:0)**	Medium Chain Fatty Acid	Lipid	**2.16**	0.0001	0.0065
**5Alpha-Pregnan-3Beta,20Beta-Diol Monosulfate (1)**	Progestin Steroids	Lipid	**2.13**	0.0053	0.0357
**Palmitoyl-Oleoyl-Glycerol (16:0/18:1)**	Diacylglycerol	Lipid	**2.12**	0.0168	0.0614
**13-Hode + 9-Hode**	Fatty Acid, Monohydroxy	Lipid	**2.11**	0.0146	0.057
**5,6-Dihydrouracil**	Pyrimidine Metabolism, Uracil containing	Nucleotide	**2.06**	0.0278	0.076
**Alpha-Ketoglutarate**	TCA Cycle	Energy	**2.05**	0.0106	0.0517
**3Beta-Hydroxy-5-Cholestenoate**	Sterol	Lipid	**2.03**	0.0147	0.057
**1-Stearoyl-2-Docosahexaenoyl-Gpe (18:0/22:6)**	Phosphatidylethanolamine (PE)	Lipid	**2.03**	0.0363	0.0858
**Behenate (22:0)**	Long Chain Fatty Acid	Lipid	**2**	0.003	0.0271
**Diacylglycerol (14:0/18:1, 16:0/16:1)**	Diacylglycerol	Lipid	**2**	0.0271	0.076
**Pyruvate**	Glycolysis, Gluconeogenesis, and Pyruvate Metabolism	Carbohydrate	**1.98**	0.0002	0.0079
**1-Palmitoleoyl-2-Linolenoyl-Gpc (16:1/18:3)**	Phosphatidylcholine (PC)	Lipid	**1.98**	0.0323	0.0809
**Lactate**	Glycolysis, Gluconeogenesis, and Pyruvate Metabolism	Carbohydrate	**1.96**	0	0.0015
**Myristoyl-Linoleoyl-Glycerol (14:0/18:2)**	Diacylglycerol	Lipid	**1.96**	0.0138	0.0565
**Lactosyl-N-Palmitoyl-Sphingosine (D18:1/16:0)**	Ceramides	Lipid	**1.93**	0.0013	0.0177

**10-Nonadecenoate (19:1N9)**	Long Chain Fatty Acid	Lipid	**1.92**	0.0051	0.0357
**Glycerol**	Glycerolipid Metabolism	Lipid	**1.91**	0.0002	0.0079
**Ceramide (D18:1/17:0, D17:1/18:0)**	Ceramides	Lipid	**1.88**	0.0443	0.0934
**Cystine**	Methionine, Cysteine, SAM and Taurine Metabolism	Amino Acid	**1.87**	0.0054	0.0357
**10-Heptadecenoate (17:1N7)**	Long Chain Fatty Acid	Lipid	**1.87**	0.0027	0.0265
**Decanoylcarnitine (C10)**	Fatty Acid Metabolism(Acyl Carnitine)	Lipid	**1.86**	0.0006	0.0113
**Lactosyl-N-Behenoyl-Sphingosine (D18:1/22:0)**	Sphingolipid Metabolism	Lipid	**1.86**	0.0311	0.0797
**2,3-Dihydroxy-2-Methylbutyrate**	Leucine, Isoleucine and Valine Metabolism	Amino Acid	**1.84**	0.0211	0.0693
**Docosadienoate (22:2N6)**	Polyunsaturated Fatty Acid (n3 and n6)	Lipid	**1.84**	0.0002	0.0079
**1-Palmitoyl-2-Docosahexaenoyl-Gpe (16:0/22:6)**	Phosphatidylethanolamine (PE)	Lipid	**1.83**	0.0332	0.0811
**Nervonate (24:1N9)**	Long Chain Fatty Acid	Lipid	**1.81**	0.0015	0.0195
**Margarate (17:0)**	Long Chain Fatty Acid	Lipid	**1.81**	0.0026	0.0261
**Linolenate [Alpha Or Gamma; (18:3N3 Or 6)]**	Polyunsaturated Fatty Acid (n3 and n6)	Lipid	**1.8**	0.0145	0.057
**Tricosanoyl Sphingomyelin (D18:1/23:0)**	Sphingolipid Metabolism	Lipid	**1.8**	0.0241	0.074
**Laurylcarnitine (C12)**	Fatty Acid Metabolism(Acyl Carnitine)	Lipid	**1.78**	0.0009	0.0144
**Dihomo-Linoleate (20:2N6)**	Polyunsaturated Fatty Acid (n3 and n6)	Lipid	**1.78**	0.0048	0.0353
**Palmitoleate (16:1N7)**	Long Chain Fatty Acid	Lipid	**1.78**	0.0084	0.0461
**N-Stearoyl-Sphingosine (D18:1/18:0)**	Ceramides	Lipid	**1.78**	0.009	0.0476
**1-Palmitoleoyl-2-Linoleoyl-Gpc (16:1/18:2)**	Phosphatidylcholine (PC)	Lipid	**1.78**	0.0116	0.0532
**Caprylate (8:0)**	Medium Chain Fatty Acid	Lipid	**1.78**	0.0236	0.0734
**5-Dodecenoate (12:1N7)**	Medium Chain Fatty Acid	Lipid	**1.78**	0.0244	0.0745
**Hexadecadienoate (16:2N6)**	Polyunsaturated Fatty Acid (n3 and n6)	Lipid	**1.78**	0.0321	0.0809
**1-Stearoyl-Gpi (18:0)**	Lysophospholipid	Lipid	**1.76**	0.0031	0.0273
**Cholesterol**	Sterol	Lipid	**1.76**	0.0049	0.0353
**Octadecenedioate (C18:1-Dc)**	Fatty Acid, Dicarboxylate	Lipid	**1.75**	0.0201	0.0685
**Docosapentaenoate (N3 Dpa; 22:5N3)**	Polyunsaturated Fatty Acid (n3 and n6)	Lipid	**1.74**	0.0026	0.0261
**Sphingomyelin (D18:2/23:0, D18:1/23:1, D17:1/24:1)**	Sphingolipid Metabolism	Lipid	**1.74**	0.0056	0.0362
**1-Palmitoyl-Gpi (16:0)**	Lysophospholipid	Lipid	**1.74**	0.0166	0.061
**Caprate (10:0)**	Medium Chain Fatty Acid	Lipid	**1.74**	0.0389	0.09
**N-Stearoyl-Sphingadienine (D18:2/18:0)**	Ceramides	Lipid	**1.73**	0.0087	0.0471
**Nonadecanoate (19:0)**	Long Chain Fatty Acid	Lipid	**1.71**	0.001	0.0148
**Sphingomyelin (D18:1/21:0, D17:1/22:0, D16:1/23:0)**	Sphingolipid Metabolism	Lipid	**1.71**	0.0162	0.0601
**17-Methylstearate (I19:0)**	Fatty Acid, Branched	Lipid	**1.7**	0.0003	0.0079
**Oleate/Vaccenate (18:1)**	Long Chain Fatty Acid	Lipid	**1.7**	0.0041	0.0325
**15-Methylpalmitate (I17:0)**	Fatty Acid, Branched	Lipid	**1.7**	0.005	0.0356
**Oleoylcarnitine (C18:1)**	Fatty Acid Metabolism(Acyl Carnitine)	Lipid	**1.7**	0.0068	0.0416
**Glycosyl-N-Behenoyl-Sphingadienine (D18:2/22:0)**	Ceramides	Lipid	**1.69**	0.0449	0.0934
**13-Methylmyristate (I15:0)**	Fatty Acid, Branched	Lipid	**1.67**	0.0076	0.0435
**Linoleate (18:2N6)**	Polyunsaturated Fatty Acid (n3 and n6)	Lipid	**1.67**	0.0104	0.0513
**Carotene Diol**	Vitamin A Metabolism	Cofactors and Vitamins	**1.66**	0.027	0.076
**5-Dodecenoylcarnitine (C12:1)**	Fatty Acid Metabolism(Acyl Carnitine)	Lipid	**1.66**	0.0011	0.015
**Arachidate (20:0)**	Long Chain Fatty Acid	Lipid	**1.66**	0.0037	0.0318
**Myristate (14:0)**	Long Chain Fatty Acid	Lipid	**1.65**	0.0141	0.0565

**1-(1-Enyl-Palmitoyl)-2-Linoleoyl-Gpc (P-16:0/18:2)**	Plasmalogen	Lipid	**1.65**	0.014	0.0565
**1-Stearoyl-2-Arachidonoyl-Gpe (18:0/20:4)**	Phosphatidylethanolamine (PE)	Lipid	**1.65**	0.0207	0.0693
**Pregnanediol-3-Glucuronide**	Progestin Steroids	Lipid	**1.65**	0.045	0.0934
**Imidazole Lactate**	Histidine Metabolism	Amino Acid	**1.64**	0.0115	0.0532
**1-Myristoyl-2-Arachidonoyl-Gpc (14:0/20:4)**	Phosphatidylcholine (PC)	Lipid	**1.64**	0.0201	0.0685
**Oleoyl-Arachidonoyl-Glycerol (18:1/20:4)**	Diacylglycerol	Lipid	**1.64**	0.0283	0.076
**Carotene Diol**	Vitamin A Metabolism	Cofactors and Vitamins	**1.63**	0.0266	0.0759
**3-Hydroxylaurate**	Fatty Acid, Monohydroxy	Lipid	**1.63**	0.0274	0.076
**Ceramide (D18:1/20:0, D16:1/22:0, D20:1/18:0)**	Ceramides	Lipid	**1.61**	0.033	0.0811
**N-Behenoyl-Sphingadienine (D18:2/22:0)**	Sphingolipid Metabolism	Lipid	**1.61**	0.0435	0.0934
**Glycosyl Ceramide (D18:1/20:0, D16:1/22:0)**	Ceramides	Lipid	**1.61**	0.0457	0.0942
**1-Palmitoyl-2-Arachidonoyl-Gpe (16:0/20:4)**	Phosphatidylethanolamine (PE)	Lipid	**1.58**	0.0231	0.0734
**Sphingomyelin (D18:2/21:0, D16:2/23:0)**	Sphingolipid Metabolism	Lipid	**1.56**	0.0007	0.0113
**Sphingomyelin (D18:1/19:0, D19:1/18:0)**	Sphingolipid Metabolism	Lipid	**1.56**	0.0006	0.0113
**Octanoylcarnitine (C8)**	Fatty Acid Metabolism(Acyl Carnitine)	Lipid	**1.56**	0.0281	0.076
**Glycosyl-N-Stearoyl-Sphingosine (D18:1/18:0)**	Ceramides	Lipid	**1.55**	0.0464	0.0942
**Pentadecanoate (15:0)**	Long Chain Fatty Acid	Lipid	**1.54**	0.0029	0.027
**Ceramide (D18:2/24:1, D18:1/24:2)**	Ceramides	Lipid	**1.53**	0.0397	0.091
**5Alpha-Pregnan-3Beta-Ol,20-One Sulfate**	Progestin Steroids	Lipid	**1.52**	0.0405	0.0919
**Sphingomyelin (D17:2/16:0, D18:2/15:0)**	Sphingolipid Metabolism	Lipid	**1.51**	0.0254	0.075
**Glycosyl-N-Palmitoyl-Sphingosine (D18:1/16:0)**	Ceramides	Lipid	**1.51**	0.0305	0.0792
**N-Palmitoyl-Sphinganine (D18:0/16:0)**	Sphingolipid Metabolism	Lipid	**1.51**	0.0447	0.0934
**2-Hydroxynervonate**	Fatty Acid, Monohydroxy	Lipid	**1.5**	0.0111	0.0529
**Ceramide (D16:1/24:1, D18:1/22:1)**	Ceramides	Lipid	**1.5**	0.0429	0.0934
**Beta-Hydroxyisovalerate**	Leucine, Isoleucine and Valine Metabolism	Amino Acid	**1.49**	0.0252	0.075
**Aconitate [Cis Or Trans]**	TCA Cycle	Energy	**1.47**	0.0059	0.0377
**1-Palmitoyl-2-Linoleoyl-Gpc (16:0/18:2)**	Phosphatidylcholine (PC)	Lipid	**1.46**	0.0287	0.0766
**4-Hydroxy-2-Oxoglutaric Acid**	Fatty Acid, Dicarboxylate	Lipid	**1.46**	0.0325	0.0809
**Stearate (18:0)**	Long Chain Fatty Acid	Lipid	**1.45**	0.0022	0.0259
**Palmitate (16:0)**	Long Chain Fatty Acid	Lipid	**1.45**	0.0084	0.0461
**Cis-4-Decenoylcarnitine (C10:1)**	Fatty Acid Metabolism(Acyl Carnitine)	Lipid	**1.45**	0.0161	0.0601
**3-Hydroxy-2-Ethylpropionate**	Leucine, Isoleucine and Valine Metabolism	Amino Acid	**1.44**	0.0003	0.0079
**Glucose**	Glycolysis, Gluconeogenesis, and Pyruvate Metabolism	Carbohydrate	**1.44**	0.0025	0.0261
**Alpha-Tocopherol**	Tocopherol Metabolism	Cofactors and Vitamins	**1.41**	0.0219	0.0714
**Sphingomyelin (D18:2/14:0, D18:1/14:1)**	Sphingolipid Metabolism	Lipid	**1.41**	0.014	0.0565
**1-Palmitoyl-2-Gamma-Linolenoyl-Gpc (16:0/18:3N6)**	Phosphatidylcholine (PC)	Lipid	**1.41**	0.0204	0.0688
**1-Linoleoyl-2-Arachidonoyl-Gpc (18:2/20:4N6)**	Phosphatidylcholine (PC)	Lipid	**1.39**	0.0363	0.0858
**Thyroxine**	Tyrosine Metabolism	Amino Acid	**1.38**	0.015	0.0572
**1-Stearoyl-2-Arachidonoyl-Gpi (18:0/20:4)**	Phosphatidylinositol (PI)	Lipid	**1.37**	0.043	0.0934
**Sphingomyelin (D18:2/18:1)**	Sphingolipid Metabolism	Lipid	**1.36**	0.002	0.024
**9-Hydroxystearate**	Fatty Acid, Monohydroxy	Lipid	**1.36**	0.0303	0.0792
**Sphingomyelin (D17:1/16:0, D18:1/15:0, D16:1/17:0)**	Sphingolipid Metabolism	Lipid	**1.34**	0.0134	0.0565
**Behenoyl Sphingomyelin (D18:1/22:0)**	Sphingolipid Metabolism	Lipid	**1.33**	0.0237	0.0734

**1-Stearoyl-2-Linoleoyl-Gpc (18:0/18:2)**	Phosphatidylcholine (PC)	Lipid	**1.33**	0.0325	0.0809
**Lignoceroyl Sphingomyelin (D18:1/24:0)**	Sphingolipid Metabolism	Lipid	**1.32**	0.017	0.0615
**Sphingomyelin (D18:2/23:1)**	Sphingolipid Metabolism	Lipid	**1.3**	0.0039	0.0318
**1-Palmitoyl-2-Oleoyl-Gpc (16:0/18:1)**	Phosphatidylcholine (PC)	Lipid	**1.28**	0.0013	0.0177
**Sphingomyelin (D18:1/24:1, D18:2/24:0)**	Sphingolipid Metabolism	Lipid	**1.28**	0.0028	0.027
**Sphingomyelin (D18:1/14:0, D16:1/16:0)**	Sphingolipid Metabolism	Lipid	**1.27**	0.0124	0.0547
**Sphingomyelin (D18:1/17:0, D17:1/18:0, D19:1/16:0)**	Sphingolipid Metabolism	Lipid	**1.24**	0.004	0.0318
**1-Oleoyl-2-Docosahexaenoyl-Gpc (18:1/22:6)**	Phosphatidylcholine (PC)	Lipid	**1.24**	0.0066	0.0414
**Palmitoyl Sphingomyelin (D18:1/16:0)**	Sphingolipid Metabolism	Lipid	**1.23**	0.0138	0.0565
**3-Methyl-2-Oxobutyrate**	Leucine, Isoleucine and Valine Metabolism	Amino Acid	**1.21**	0.0236	0.0734
**Sphingomyelin (D18:1/22:2, D18:2/22:1, D16:1/24:2)**	Sphingolipid Metabolism	Lipid	**1.21**	0.0331	0.0811
**Citrate**	TCA Cycle	Energy	**1.2**	0.0249	0.0749
**Sphingomyelin (D18:1/20:0, D16:1/22:0)**	Sphingolipid Metabolism	Lipid	**1.2**	0.0254	0.075
**Palmitoyl Ethanolamide**	Endocannabinoid	Lipid	**1.19**	0.0093	0.0477
**Sphingomyelin (D18:2/24:1, D18:1/24:2)**	Sphingolipid Metabolism	Lipid	**1.14**	0.0073	0.0424
**1-Palmitoyl-2-Palmitoleoyl-Gpc (16:0/16:1)**	Phosphatidylcholine (PC)	Lipid	**1.14**	0.04	0.0912
**Arginine**	Urea cycle; Arginine and Proline Metabolism	Amino Acid	**0.8**	0.0444	0.0934
**Trans-4-Hydroxyproline**	Urea cycle; Arginine and Proline Metabolism	Amino Acid	**0.77**	0.0289	0.0768
**Histidine**	Histidine Metabolism	Amino Acid	**0.76**	0.0156	0.0587
**Serine**	Glycine, Serine and Threonine Metabolism	Amino Acid	**0.75**	0.0201	0.0685
**Citrulline**	Urea cycle; Arginine and Proline Metabolism	Amino Acid	**0.73**	0.0081	0.0457
**Threonine**	Glycine, Serine and Threonine Metabolism	Amino Acid	**0.72**	0.0275	0.076
**Ornithine**	Urea cycle; Arginine and Proline Metabolism	Amino Acid	**0.69**	0.0348	0.0839
**Tryptophan**	Tryptophan Metabolism	Amino Acid	**0.69**	0.0493	0.0997
**Indole-3-Carboxylic Acid**	Tryptophan Metabolism	Amino Acid	**0.66**	0.0078	0.0445
**Tetradecanedioate (C14-Dc)**	Fatty Acid, Dicarboxylate	Lipid	**0.66**	0.0281	0.076
**Lysine**	Lysine Metabolism	Amino Acid	**0.64**	0.0053	0.0357
**S-1-Pyrroline-5-Carboxylate**	Glutamate Metabolism	Amino Acid	**0.63**	0.0393	0.0905
**Picolinate**	Tryptophan Metabolism	Amino Acid	**0.61**	0.0135	0.0565
**Hexadecenedioate (C16:1-Dc)**	Fatty Acid, Dicarboxylate	Lipid	**0.61**	0.0098	0.0491
**Gamma-Glutamyl-Alpha-Lysine**	Gamma-glutamyl Amino Acid	Peptide	**0.59**	0.0039	0.0318
**Indolebutyrate**	Tryptophan Metabolism	Amino Acid	**0.58**	0.0444	0.0934
**5-Methyluridine (Ribothymidine)**	Pyrimidine Metabolism, Uracil containing	Nucleotide	**0.58**	0.0005	0.0104
**Bilirubin (Z,Z)**	Hemoglobin and Porphyrin Metabolism	Cofactors and Vitamins	**0.57**	0.015	0.0572
**N-Palmitoylserine**	Endocannabinoid	Lipid	**0.56**	0.0419	0.0934
**Homoarginine**	Urea cycle; Arginine and Proline Metabolism	Amino Acid	**0.51**	0.021	0.0693
**Phosphoethanolamine**	Phospholipid Metabolism	Lipid	**0.5**	0.0255	0.075
**Glycohyocholate**	Secondary Bile Acid Metabolism	Lipid	**0.5**	0.0443	0.0934
**Bilirubin (E,Z Or Z,E)**	Hemoglobin and Porphyrin Metabolism	Cofactors and Vitamins	**0.46**	0.0261	0.0759
**Bilirubin (E,E)**	Hemoglobin and Porphyrin Metabolism	Cofactors and Vitamins	**0.39**	0.0095	0.0479
**Androstenediol (3Beta,17Beta) Disulfate (2)**	Androgenic Steroids	Lipid	**0.37**	0.0422	0.0934
**Pyridoxal**	Vitamin B6 Metabolism	Cofactors and Vitamins	**0.34**	0.0453	0.0937
**Adenosine**	Purine Metabolism, Adenine containing	Nucleotide	**0.33**	0.0228	0.0731

**Estriol 3-Sulfate**	Estrogenic Steroids	Lipid	**0.32**	0.0189	0.0657
**17Alpha-Hydroxypregnenolone 3-Sulfate**	Pregnenolone Steroids	Lipid	**0.31**	0.0266	0.0759
**Androstenediol (3Beta,17Beta) Disulfate (1)**	Androgenic Steroids	Lipid	**0.31**	0.0464	0.0942
**N-Acetylkynurenine**	Tryptophan Metabolism	Amino Acid	**0.3**	0.0145	0.057
**21-Hydroxypregnenolone Monosulfate**	Pregnenolone Steroids	Lipid	**0.3**	0.0278	0.076
**N-Acetyl-2-Aminooctanoate**	Fatty Acid, Amino	Lipid	**0.27**	0.0031	0.0273
**Andro Steroid Monosulfate C19H28O6S**	Androgenic Steroids	Lipid	**0.26**	0.0174	0.0619
**16A-Hydroxy Dhea 3-Sulfate**	Androgenic Steroids	Lipid	**0.22**	0.0071	0.042
**3-Dehydrocholate**	Secondary Bile Acid Metabolism	Lipid	**0.13**	0.0022	0.0259

Table S3. Fold-change in cord plasma (VC/EC) for xenobiotics with significant *p* (≤0.05) and *q* (≤0.10), ordered according to fold-change (greatest to smallest), with corresponding sub-pathway and super-pathway (of note, the fold-change for Metronidazole is recorded as 0 as there was 0 quantity recorded in the VC group, however the *p*-value is the smallest).

Metabolite	Sub-Pathway	Super-Pathway	Fold-Change (VC/EC)	*p*-Value	*q*-Value
**Triethanolamine**	Chemical	Xenobiotics	**39.65**	0.0028	0.0298
**4-Acetamidophenol**	Drug - Analgesics, Anesthetics	Xenobiotics	**16.03**	0.0000	0.0031
**4-Acetaminophen Sulfate**	Drug - Analgesics, Anesthetics	Xenobiotics	**7.73**	0.0004	0.0093
**4-Acetamidophenylglucuronide**	Drug - Analgesics, Anesthetics	Xenobiotics	**5.60**	0.0003	0.0093
**2-Hydroxyacetaminophen Sulfate**	Drug - Analgesics, Anesthetics	Xenobiotics	**5.60**	0.0005	0.0100
**2-Methoxyacetaminophen Sulfate**	Drug - Analgesics, Anesthetics	Xenobiotics	**5.09**	0.0002	0.0081
**2-Methoxyacetaminophen Glucuronide**	Drug - Analgesics, Anesthetics	Xenobiotics	**4.13**	0.0004	0.0093
**3-(Cystein-S-Yl)Acetaminophen**	Drug - Analgesics, Anesthetics	Xenobiotics	**3.99**	0.0005	0.0099
**3-(N-Acetyl-L-Cystein-S-Yl) Acetaminophen**	Drug - Analgesics, Anesthetics	Xenobiotics	**3.71**	0.0087	0.0526
**Ibuprofen**	Drug - Analgesics, Anesthetics	Xenobiotics	**2.71**	0.0011	0.0146
**Benzoate**	Benzoate Metabolism	Xenobiotics	**2.26**	0.0158	0.0734
**Ranitidine**	Drug - Gastrointestinal	Xenobiotics	**0.54**	0.0059	0.0413
**Acesulfame**	Food Component/Plant	Xenobiotics	**0.31**	0.0061	0.0424
**Saccharin**	Food Component/Plant	Xenobiotics	**0.04**	0.0000	0.0000
**Metronidazole**	Drug - Antibiotic	Xenobiotics	**0.00**	0.0000	0.0000

Table S4. Fold-change in maternal (intervillous) plasma (VM/EM) for xenobiotics with significant *p* (≤0.05) and *q* (≤0.10), ordered according to fold-change (greatest to smallest) (of note, the fold-change for Metronidazole is recorded as 0 as there was 0 quantity recorded in the VC group, however the *p*-value is the smallest).

Metabolite	Sub-Pathway	Super-Pathway	Fold-Change (VM/EM)	*p*-Value	*q*-Value
**Triethanolamine**	Chemical	Xenobiotics	**19.54**	0.0121	0.0539
**4-Acetamidophenol**	Drug - Analgesics, Anesthetics	Xenobiotics	**18.20**	0.0000	0.0039
**3-(Cystein-S-Yl) Acetaminophen**	Drug - Analgesics, Anesthetics	Xenobiotics	**10.15**	0.0007	0.0113
**4-Acetaminophen Sulfate**	Drug - Analgesics, Anesthetics	Xenobiotics	**10.09**	0.0002	0.0079
**2-Methoxyacetaminophen Sulfate**	Drug - Analgesics, Anesthetics	Xenobiotics	**9.91**	0.0001	0.0066
**4-Acetamidophenylglucuronide**	Drug - Analgesics, Anesthetics	Xenobiotics	**8.84**	0.0002	0.0078
**3-(N-Acetyl-L-Cystein-S-Yl) Acetaminophen**	Drug - Analgesics, Anesthetics	Xenobiotics	**8.27**	0.0070	0.0417
**2-Hydroxyacetaminophen Sulfate**	Drug - Analgesics, Anesthetics	Xenobiotics	**8.06**	0.0003	0.0079
**2-Methoxyacetaminophen Glucuronide**	Drug - Analgesics, Anesthetics	Xenobiotics	**7.39**	0.0001	0.0070
**4-Hydroxybenzoate**	Benzoate Metabolism	Xenobiotics	**6.14**	0.0107	0.0517
**Morphine-6-Glucuronide**	Drug - Analgesics, Anesthetics	Xenobiotics	**3.91**	0.0370	0.0870
**Morphine-3-Glucuronide**	Drug - Analgesics, Anesthetics	Xenobiotics	**3.05**	0.0380	0.0885
**Ibuprofen**	Drug - Analgesics, Anesthetics	Xenobiotics	**2.90**	0.0004	0.0082
**Benzoate**	Benzoate Metabolism	Xenobiotics	**2.78**	0.0444	0.0934
**Salicyluric Glucuronide**	Drug - Analgesics, Anesthetics	Xenobiotics	**2.75**	0.0000	0.0003
**Phytanate**	Food Component/Plant	Xenobiotics	**2.51**	0.0025	0.0261
**3-Methylxanthine**	Xanthine Metabolism	Xenobiotics	**2.19**	0.0233	0.0734
**7-Methylxanthine**	Xanthine Metabolism	Xenobiotics	**1.78**	0.0247	0.0748
**Umbelliferone Sulfate**	Food Component/Plant	Xenobiotics	**0.32**	0.0376	0.0878
**Saccharin**	Food Component/Plant	Xenobiotics	**0.14**	0.0007	0.0113
**Metronidazole**	Drug - Antibiotic	Xenobiotics	**0.00**	0.0000	0.0000

Table S5. Fold-change in cord plasma (VC/EC) and maternal (intervillous) plasma (VM/EM) for sphingolipids, with corresponding *p* and *q* values (significant when *p* ≤ 0.05 and *q* ≤ 0.10).

Sphingolipid	Fold-Change (VC/EC)	*p*-Value	*q*-Value	Fold-Change (VM/EM)	*p*-Value	*q*-Value
**Sphinganine**	**1.54**	NS	0.3393	**1.28**	NS	0.362
**Sphinganine-1-Phosphate**	**1.28**	NS	0.2858	**1.53**	NS	0.197
**N-Palmitoyl-Sphinganine (D18:0/16:0)**	**1.42**	NS	0.1602	**1.51**	0.045	0.093
**N-Palmitoyl-Sphingadienine (D18:2/16:0)**	**1.21**	NS	0.494	**1.28**	NS	0.213
**N-Behenoyl-Sphingadienine (D18:2/22:0)**	**1.06**	NS	0.5461	**1.61**	0.044	0.093
**Myristoyl Dihydrosphingomyelin (D18:0/14:0)**	**1.17**	NS	0.216	**1.02**	NS	0.498
**Palmitoyl Dihydrosphingomyelin (D18:0/16:0)**	**1.04**	NS	0.4674	**0.98**	NS	0.452
**Behenoyl Dihydrosphingomyelin (D18:0/22:0)**	**1.06**	NS	0.5635	**0.9**	NS	0.261
**Palmitoyl Sphingomyelin (D18:1/16:0)**	**1.06**	NS	0.2737	**1.23**	0.014	0.057
**Stearoyl Sphingomyelin (D18:1/18:0)**	**0.9**	NS	0.3165	**1.02**	NS	0.466

**Behenoyl Sphingomyelin (D18:1/22:0)**	**0.94**	NS	0.4308	**1.33**	0.024	0.073
**Tricosanoyl Sphingomyelin (D18:1/23:0)**	**1.05**	NS	0.5663	**1.8**	0.024	0.074
**Lignoceroyl Sphingomyelin (D18:1/24:0)**	**1.06**	NS	0.5133	**1.32**	0.017	0.062
**Sphingomyelin (D18:1/14:0, D16:1/16:0)**	**1.05**	NS	0.4497	**1.27**	0.012	0.055
**Sphingomyelin (D18:2/14:0, D18:1/14:1)**	**1.08**	NS	0.4942	**1.41**	0.014	0.057
**Sphingomyelin (D17:1/16:0, D18:1/15:0, D16:1/17:0)**	**1.12**	NS	0.2597	**1.34**	0.013	0.057
**Sphingomyelin (D18:2/16:0, D18:1/16:1)**	**1.03**	NS	0.5712	**1.09**	NS	0.118
**Sphingomyelin (D18:1/17:0, D17:1/18:0, D19:1/16:0)**	**1.09**	NS	0.3643	**1.24**	0.004	0.032
**Sphingomyelin (D18:1/18:1, D18:2/18:0)**	**0.98**	NS	0.5218	**0.88**	NS	0.335
**Sphingomyelin (D18:1/20:0, D16:1/22:0)**	**0.92**	NS	0.3642	**1.2**	0.025	0.075
**Sphingomyelin (D18:1/20:1, D18:2/20:0)**	**0.93**	NS	0.4066	**0.91**	NS	0.388
**Sphingomyelin (D18:1/21:0, D17:1/22:0, D16:1/23:0)**	**1.14**	NS	0.4942	**1.71**	0.016	0.06
**Sphingomyelin (D18:1/22:1, D18:2/22:0, D16:1/24:1)**	**1**	NS	0.5663	**1.09**	NS	0.351
**Sphingomyelin (D18:2/23:0, D18:1/23:1, D17:1/24:1)**	**1.28**	NS	0.1998	**1.74**	0.006	0.036
**Sphingomyelin (D18:1/24:1, D18:2/24:0)**	**1.06**	NS	0.4865	**1.28**	0.003	0.027
**Sphingomyelin (D18:2/24:1, D18:1/24:2)**	**1**	NS	0.579	**1.14**	0.007	0.042
**Sphingosine**	**1.83**	NS	0.1642	**0.98**	NS	0.539
**Sphingosine 1-Phosphate**	**1.27**	NS	0.1783	**1.38**	NS	0.109
**Sphingomyelin (D18:2/23:1)**	**1.12**	NS	0.3657	**1.3**	0.004	0.032
**Sphingomyelin (D18:2/21:0, D16:2/23:0)**	**1.23**	NS	0.2532	**1.56**	0.001	0.011
**Sphingomyelin (D18:1/20:2, D18:2/20:1, D16:1/22:2)**	**1.06**	NS	0.5133	**1.18**	NS	0.208
**Sphingomyelin (D18:2/24:2)**	**0.89**	NS	0.2953	**0.94**	NS	0.468
**Sphingomyelin (D18:1/25:0, D19:0/24:1, D20:1/23:0, D19:1/24:0)**	**1.64**	0.046	0.1299	**1.14**	NS	0.314
**Sphingomyelin (D18:1/22:2, D18:2/22:1, D16:1/24:2)**	**0.92**	NS	0.3643	**1.21**	0.033	0.081
**Sphingomyelin (D18:0/20:0, D16:0/22:0)**	**1.16**	NS	0.5509	**0.92**	NS	0.309
**Sphingomyelin (D18:0/18:0, D19:0/17:0)**	**1.01**	NS	0.5748	**0.81**	NS	0.22
**Sphingomyelin (D17:2/16:0, D18:2/15:0)**	**1.09**	NS	0.4276	**1.51**	0.025	0.075
**Sphingomyelin (D18:2/18:1)**	**1.07**	NS	0.4308	**1.36**	0.002	0.024
**Sphingomyelin (D18:1/19:0, D19:1/18:0)**	**1.09**	NS	0.4352	**1.56**	0.001	0.011
**N-Palmitoyl-Heptadecasphingosine (D17:1/16:0)**	**1.15**	NS	0.4012	**1.54**	NS	0.188
**N-Stearoyl-Sphinganine (D18:0/18:0)**	**1.12**	NS	0.5295	**1.16**	NS	0.49
**Sphingadienine**	**1.35**	NS	0.5274	**0.58**	NS	0.264
**Lactosyl-N-Behenoyl-Sphingosine (D18:1/22:0)**	**1.94**	NS	0.2146	**1.86**	0.031	0.08

Table S6. Fold-change in cord plasma (VC/EC) and maternal (intervillous) plasma (VM/EM) for pregnenolone steroids, with corresponding *p* and *q* values (significant when $p \leq 0.05$ and $q \leq 0.10$).

Pregnenolone Steroids	Fold-Change (VC/EC)	*p*-Value	*q*-Value	Fold-Change (VM/EM)	*p*-Value	*q*-Value
**Pregnenolone Sulfate**	**1.10**	NS	0.5121	**0.39**	0.055	0.108
**17alpha-Hydroxypregnenolone 3-Sulfate**	**1.23**	NS	0.5178	**0.31**	0.027	0.076
**17alpha-Hydroxypregnanolone Glucuronide**	**0.85**	NS	0.2681	**0.9**	NS	0.435
**21-Hydroxypregnenolone Monosulfate**	**0.78**	NS	0.2146	**0.3**	0.028	0.076
**21-Hydroxypregnenolone Disulfate**	**0.94**	NS	0.5039	**0.44**	NS	0.15
**21-Hydroxypregnanolone Disulfate**	**1.12**	NS	0.4033	**1**	NS	0.417
**Pregnen-Diol Disulfate C21H34O8S2**	**1.37**	NS	0.5635	**1.01**	NS	0.47
**Pregn Steroid Monosulfate C21H34O5S**	**1.41**	NS	0.3514	**0.86**	NS	0.451

Table S7. Fold-change in cord plasma (VC/EC) and maternal (intervillous) plasma (VM/EM) for progestin steroids, with corresponding *p* and *q* values (significant when $p \leq 0.05$ and $q \leq 0.10$).

Progestin Steroids	Fold-Change (VC/EC)	*p*-Value	Q-Value	Fold-Change (VM/EM)	*p*-Value	*q*-Value
**Progesterone**	**1.42**	NS	0.1545	**1.38**	NS	0.532
**5alpha-Pregnan-3beta-Ol,20-One Sulfate**	**1.11**	NS	0.4856	**1.52**	0.041	0.092
**5alpha-Pregnan-3beta,20beta-Diol Monosulfate**	**1.34**	NS	0.3011	**2.13**	0.005	0.036
**5alpha-Pregnan-3beta,20alpha-Diol Monosulfate 1**	**1.36**	NS	0.2510	**2.31**	0.005	0.035
**5alpha-Pregnan-3beta,20alpha-Diol Monosulfate 2**	**1.52**	NS	0.3514	**2.25**	0.004	0.032
**5alpha-Pregnan-3beta,20alpha-Diol Disulfate**	**1.12**	NS	0.3907	**1.34**	NS	0.309
**5alpha-pregnan-diol disulfate**	**1.05**	NS	0.4194	**1.69**	NS	0.3101
**5alpha-Pregnan-3alpha,20beta-Diol Disulfate**	**1.43**	NS	0.1853	**0.86**	NS	0.314
**Pregnanediol-3-Glucuronide**	**0.95**	NS	0.5413	**1.65**	0.045	0.093
**Pregnanolone/Allopregnanolone Sulfate**	**1.08**	NS	0.5635	**1.67**	0.059	0.111

Table S8. Fold-change in cord plasma (VC/EC) and maternal (intervillous) plasma (VM/EM) for endocannabinoid steroids, with corresponding *p* and *q* values (significant when $p \leq 0.05$ and $q \leq 0.10$).

Endocannabinoid Steroids	Fold-Change (VC/EC)	*p*-Value	*q*-Value	Fold-Change (VM/EM)	*p*-Value	*q*-Value
**Palmitoyl Ethanolamide (PEA)**	**1.19**	0.0357	0.1158	**1.19**	0.009	0.048
**N-Palmitoylserine**	**0.93**	NS	0.4460	**0.56**	0.042	0.093
**Oleoyl Ethanolamide (OEA)**	**1.31**	0.0226	0.0904	**1.39**	NS	0.209
**Arachidonoyl Ethanolamide (AEA/Andamide)**	**1.48**	0.0296	0.1015	**1.25**	NS	0.277
**Linoleoyl Ethanolamide**	**1.34**	0.0707	0.1642	**1.32**	NS	0.226
**N-Stearoyltaurine**	**0.96**	NS	0.5605	**1.22**	NS	0.413
**N-Palmitoyltaurine**	**0.97**	NS	0.5321	**0.81**	NS	0.255
**N-Linoleoyltaurine**	**0.98**	NS	0.5461	**0.83**	NS	0.196
**N-Oleoylserine**	**0.94**	NS	0.5133	**0.77**	NS	0.311

Table S9. Fold-change in cord plasma (VC/EC) and maternal (intervillous) plasma (VM/EM) for ceramides, with corresponding *p* and *q* values (significant when $p \leq 0.05$ and $q \leq 0.10$).

Ceramide	Fold-Change (VC/EC)	*p*-Value	*q*-Value	Fold-Change (VM/EM)	*p*-Value	*q*-Value
**N-Palmitoyl-Sphingosine (D18:1/16:0)**	**1.38**	NS	0.1455	**1.49**	NS	0.135
**N-Stearoyl-Sphingosine (D18:1/18:0)**	**1.27**	NS	0.3510	**1.78**	0.009	0.048
**N-Stearoyl-Sphingadienine (D18:2/18:0)**	**1.49**	NS	0.1491	**1.73**	0.009	0.047
**Ceramide (D16:1/24:1, D18:1/22:1)**	**1.26**	NS	0.3228	**1.5**	0.043	0.093
**Ceramide (D18:1/14:0, D16:1/16:0)**	**1.28**	NS	0.3303	**1.53**	NS	0.119
**Ceramide (D18:1/17:0, D17:1/18:0)**	**1.79**	NS	0.1950	**1.88**	0.044	0.093
**Ceramide (D18:1/20:0, D16:1/22:0, D20:1/18:0)**	**1.05**	NS	0.5688	**1.61**	0.033	0.081
**Ceramide (D18:2/24:1, D18:1/24:2)**	**1.34**	NS	0.2513	**1.53**	0.04	0.091
**Glycosyl-N-Palmitoyl-Sphingosine (D18:1/16:0)**	**1.35**	NS	0.1674	**1.51**	0.031	0.079
**Glycosyl-N-Stearoyl-Sphingosine (D18:1/18:0)**	**1.21**	NS	0.4308	**1.55**	0.046	0.094
**Glycosyl-N-Behenoyl-Sphingadienine (D18:2/22:0)**	**1.38**	NS	0.4688	**1.69**	0.045	0.093
**Glycosyl-N-(2-Hydroxynervonoyl)-Sphingosine (D18:1/24:1(2OH))**	**0.57**	NS	0.5276	**1.99**	NS	0.121
**Lactosyl-N-Palmitoyl-Sphingosine (D18:1/16:0)**	**1.73**	NS	0.1461	**1.93**	0.001	0.018
**Lactosyl-N-Nervonoyl-Sphingosine (D18:1/24:1)**	**2.99**	NS	0.1762	**2.83**	0.002	0.026
**Glycosyl Ceramide (D18:1/20:0, D16:1/22:0)**	**1.22**	NS	0.5114	**1.61**	0.046	0.094
**Glycosyl Ceramide (D18:1/23:1, D17:1/24:1)**	**1.67**	NS	0.3228	**1.36**	NS	0.324
**Glycosyl Ceramide (D18:2/24:1, D18:1/24:2)**	**1.33**	NS	0.3755	**1.5**	NS	0.112

© 2019 by the authors. Submitted for possible open access publication under the terms and conditions of the Creative Commons Attribution (CC BY) license (http://creativecommons.org/licenses/by/4.0/).
